# M-Ionic: prediction of metal-ion-binding sites from sequence using residue embeddings

**DOI:** 10.1093/bioinformatics/btad782

**Published:** 2024-01-04

**Authors:** Aditi Shenoy, Yogesh Kalakoti, Durai Sundar, Arne Elofsson

**Affiliations:** Science for Life Laboratory and Department of Biochemistry and Biophysics, Stockholm University, Solna 17121, Sweden; Department of Biochemical Engineering & Biotechnology, Indian Institute of Technology (IIT) Delhi, New Delhi 110016, India; Department of Biochemical Engineering & Biotechnology, Indian Institute of Technology (IIT) Delhi, New Delhi 110016, India; Yardi School of Artificial Intelligence, Indian Institute of Technology (IIT) Delhi, New Delhi 110016, India; Science for Life Laboratory and Department of Biochemistry and Biophysics, Stockholm University, Solna 17121, Sweden

## Abstract

**Motivation:**

Understanding metal–protein interaction can provide structural and functional insights into cellular processes. As the number of protein sequences increases, developing fast yet precise computational approaches to predict and annotate metal-binding sites becomes imperative. Quick and resource-efficient pre-trained protein language model (pLM) embeddings have successfully predicted binding sites from protein sequences despite not using structural or evolutionary features (multiple sequence alignments). Using residue-level embeddings from the pLMs, we have developed a sequence-based method (M-Ionic) to identify metal-binding proteins and predict residues involved in metal binding.

**Results:**

On independent validation of recent proteins, M-Ionic reports an area under the curve (AUROC) of 0.83 (recall = 84.6%) in distinguishing metal binding from non-binding proteins compared to AUROC of 0.74 (recall = 61.8%) of the next best method. In addition to comparable performance to the state-of-the-art method for identifying metal-binding residues (Ca^2+^, Mg^2+^, Mn^2+^, Zn^2+^), M-Ionic provides binding probabilities for six additional ions (i.e. Cu^2+^, ${\text{Po}}_{4}^{3-}$, ${\text{So}}_{4}^{2-}$, Fe^2+^, Fe^3+^, Co^2+^). We show that the pLM embedding of a single residue contains sufficient information about its neighbours to predict its binding properties.

**Availability and implementation:**

M-Ionic can be used on your protein of interest using a Google Colab Notebook (https://bit.ly/40FrRbK). The GitHub repository (https://github.com/TeamSundar/m-ionic) contains all code and data.

## 1 Introduction

Proteins containing metal cofactors (metalloproteins) are involved in various cellular biochemical processes, including protein folding (Palm-Espling *et al.* 2012), enzymatic reactions (Andreini *et al.* 2009), and signalling (Maret 2017). Understanding metal-binding specificity can provide information about the functions and mechanisms of proteins. Of all proteins, about one-third of them are known to interact with metal ions (Tainer *et al.* 1991, Andreini *et al.* 2009). However, as per (Aptekmann *et al.* 2022), only ∼14% is annotated as metal binding in the UniProt database (The UniProt Consortium 2017). The size of metal ions, protein activation by a variety of ions (metal promiscuity (Eom and Song 2019)), and protein precipitation while dealing with high concentrations of samples (Witkowska and Rowińska-Żyrek 2019) are some of the difficulties involved with experimental methods making them labour-intensive and time-consuming.

Multiple attempts to develop *in silico* alternatives for identifying metal-ion-binding sites have been previously proposed. These prediction methods can be broadly categorized into three categories, namely (i) sequence-based, (ii) structure-based, and (iii) both sequence/structure-based based on the features used for training. Structure-based methods such as MIB2 (Lu *et al.* 2022) consider the three-dimensional neighbourhood surrounding the metal ion. While sequence-based methods like LMetalSite (Yuan *et al.* 2022b), mebi-pred (Aptekmann *et al.* 2022), and IonSeq (Hu *et al.* 2016) make use of the protein sequence for training the estimator, IonCon (Hu *et al.* 2016) employs both sequence and structural information. However, these methods are limited by the availability of template structures and coverage of essential metal ions. Embeddings extracted from various protein language models (pLMs), such as ESM-1b (Rives *et al.* 2021) and ProtTrans (Elnaggar *et al.* 2022), are quick to generate and have shown good performance for identifying subcellular localization (Stärk *et al.* 2021), post-translational modification (Pokharel *et al.* 2022) and ligand binding sites (Littmann *et al.* 2021).

In this study, residue-level pLM embeddings were employed to estimate (i) the likelihood of a given metal ion binding to a protein (protein level prediction) and (ii) the probability of individual protein residues interacting with the given metal ion (residue level prediction). M-Ionic provides binding-site predictions for the ten most frequently occurring metal ion groups with residue-level binding probabilities for multiple ions simultaneously. Despite using only single residue embedding, M-Ionic performs comparably to recent metal-binding predictors such as mebi-pred (Aptekmann *et al.* 2022) and LMetalSite (Yuan et al. 2022b). Therefore, we show that residue embeddings can effectively predict metal-ion-binding sites.

## 2 Materials and methods

### 2.1 Datasets

In this study, we have used three sets of metal-binding proteins. As in earlier studies (Hu *et al.* 2016, Cao *et al.* 2017, Yuan *et al.* 2022b), proteins from BioLip were used for training. Secondly, two independent sets were used for validation, one consisting of newer proteins in BioLip and the benchmark set from MIonSite. Finally, we also created a negative set from PDB. These are all described in detail below.

#### 2.1.1 Training, validation, and independent test set

Metal-binding proteins (*n* = 29 404) were curated from the BioLip database (Yang *et al.* 2013) on 2022–09-27. Proteins having sequence length >1000, resolution (>3Å), and ions with less than 1000 protein entries were removed (Supplementary Table S1). Sequences were clustered using CD-HIT (Li and Godzik 2006) at 100% sequence identity to remove identical sequences with contradictory labels. Previous studies (Krissinel 2007, Pearson 2013) have shown that homology exists between proteins having 30% sequence identity. Hence, we performed homology reduction using a sequence similarity threshold of 20% using BlastClust (Altschul *et al.* 1990) with the parameters -p T -L 0.9 -b T -S 20. The 6203 clusters obtained were shuffled and split into six partitions. One partition (TestFold6) (*n* = 3002) was reserved upfront as the independent test set. The remaining five partitions (*n* = 26 407) were used to train the estimator using a five-fold cross-validation strategy. Note: TestFold6 was not used during the training and validation of M-Ionic and had no homology with the training datasets. M-Ionic performance was further benchmarked on two independent datasets: (i) Recent BioLip (*n* = 5377) consisting of only recent proteins and (ii) MIonSite Benchmark dataset (*n* = 267) (Qiao and Xie 2019).

#### 2.1.2 Recent BioLip proteins

Proteins from the BioLip dataset (Yang *et al.* 2013) after 2022–09-27 till 2023–05-31 were used as an independent test set. Metal ions having a minimum of 1000 proteins and each having resolution (<3Å) were selected. Identical sequences (100% sequence identity threshold) were removed using CD-HIT (Li and Godzik 2006). Since these proteins are outside the training/testing datasets of M-Ionic, mebi-pred (Aptekmann *et al.* 2022), and LMetalSite (Yuan et al. 2022b), the ‘Recent BioLip’ dataset (*n* = 5377) allows for unbiased testing.

#### 2.1.3 Independent benchmark dataset from MIonSite

The performance of M-Ionic was benchmarked against other metal-binding predictors (LMetalSite (Yuan *et al.* 2022b); GASS-Metal (Paiva *et al.* 2022), IonSeq and IonCon (Hu *et al.* 2016); TargetS (Yu *et al.* 2013); MetalDetector (Lippi *et al.* 2008)) using the independent validation dataset from MIonSite (Qiao and Xie 2019). Note: The benchmark testing dataset from MIonSite (*n* = 267) differs from the M-Ionic testing set (TestFold6) (*n* = 3002) described above. The two sets have an overlap of only 10 PDB chains (5y9e_E, 5z68_B, 5z9x_A, 6c1j_A, 6c33_A, 6ci7_B, 6cko_C, 6d0y_C, 6ekb_A, 6eke_B).

#### 2.1.4 Negative metal-binding dataset

To evaluate whether the method can distinguish between (metal) binding and non-binding proteins, a negative set (i.e. protein chains that do not bind to any metal ion) was created. Protein bio-assemblies from PDB (Berman *et al.* 2000) with less than six protein chains and chains with no metals in the HETATM records were filtered (*n* = 3810) and clustered at 100% identity. The resulting dataset (*n* = 3224) was used alongside the ‘Recent BioLip’ dataset (Table 1 and Fig. 2a and b) and TestFold6 (Supplementary Table S2 and Supplementary Fig. S1a and b) to plot the ROC curves.

**Table 1.** Performance on distinguishing metal binding and non-binding proteins using the ‘Recent BioLip’ dataset and the negative set.

Methods	Prec.	Rec.	Acc.	F1	MCC	AUPR	AUROC
Mebi-pred (2022)	**0.39**	0.56	**0.84**	0.46	0.38	0.27	0.72
LMetalSite (2022)	0.38	0.62	0.83	0.47	0.39	0.28	0.74
M-Ionic (Current)	0.39	**0.85**	0.82	**0.53**	**0.49**	**0.35**	**0.83**

Bold values represent the best value in each category.

### 2.2 Feature extraction

#### 2.2.1 Sequence representation

ESM-2 (Rives *et al.* 2021) and ProtT5 (Elnaggar *et al.* 2022) residue-level embeddings were generated for all sequences. The dimension of each ESM-2 (esm2_t33_650M_UR50D) protein embedding was *Lx1280*, and that of ProtT5 (ProtT5-XL-UniRef50) protein embedding was *Lx1024*, where L is the length of the protein (i.e. each residue has a 1280 or 1024 dimension embedding).

#ESM-2

python3 esm/scripts/extract.py esm2_t33_650M_UR50D input.fasta esm_embeddings––repr_layers 33––include per_tok

#ProtT5

python3 Embedding/prott5_embedder.py––input input.fasta––output proT5_embeddings.h5

#### 2.2.2 MSA representation

Multiple sequence alignments (MSA) were generated using HHblits (Remmert *et al.* 2012) version 3.3.0 with three iterations against UniClust30 (Mirdita *et al.* 2017) (version Uniclust30_2018_08).

hhblits -i $FASTA -oa3m $OUTPUT -n 3 -d uniclust30_2018_08/uniclust30_2018_08 -cpu 32

The MSA was converted into a one-hot MSA and reweighted with a cut-off = 0.8 to generate the position-specific scoring matrices (PSSM) (Adhikari 2020). The MSAs were also converted to ESM-MSA-1b (esm_msa1b_t12_100M_UR50S) protein embedding, i.e. *Lx768* dimensional vector where L is the protein sequence length.

#### 2.2.3 Structural properties

An *Lx14* feature DSSP vector was generated as in Yuan et al. (2022a). This vector has three components: (i) One-hot encoding for eight secondary structure states along with one dimension for unknown states (*Lx9*), (ii) sine and cosine transformations of the torsion backbone phi/psi angles (*Lx4*), and (iii) relative solvent accessibility (*Lx1*), where *L* is the length of the protein.

### 2.3 Implementation

**Figure 1.**
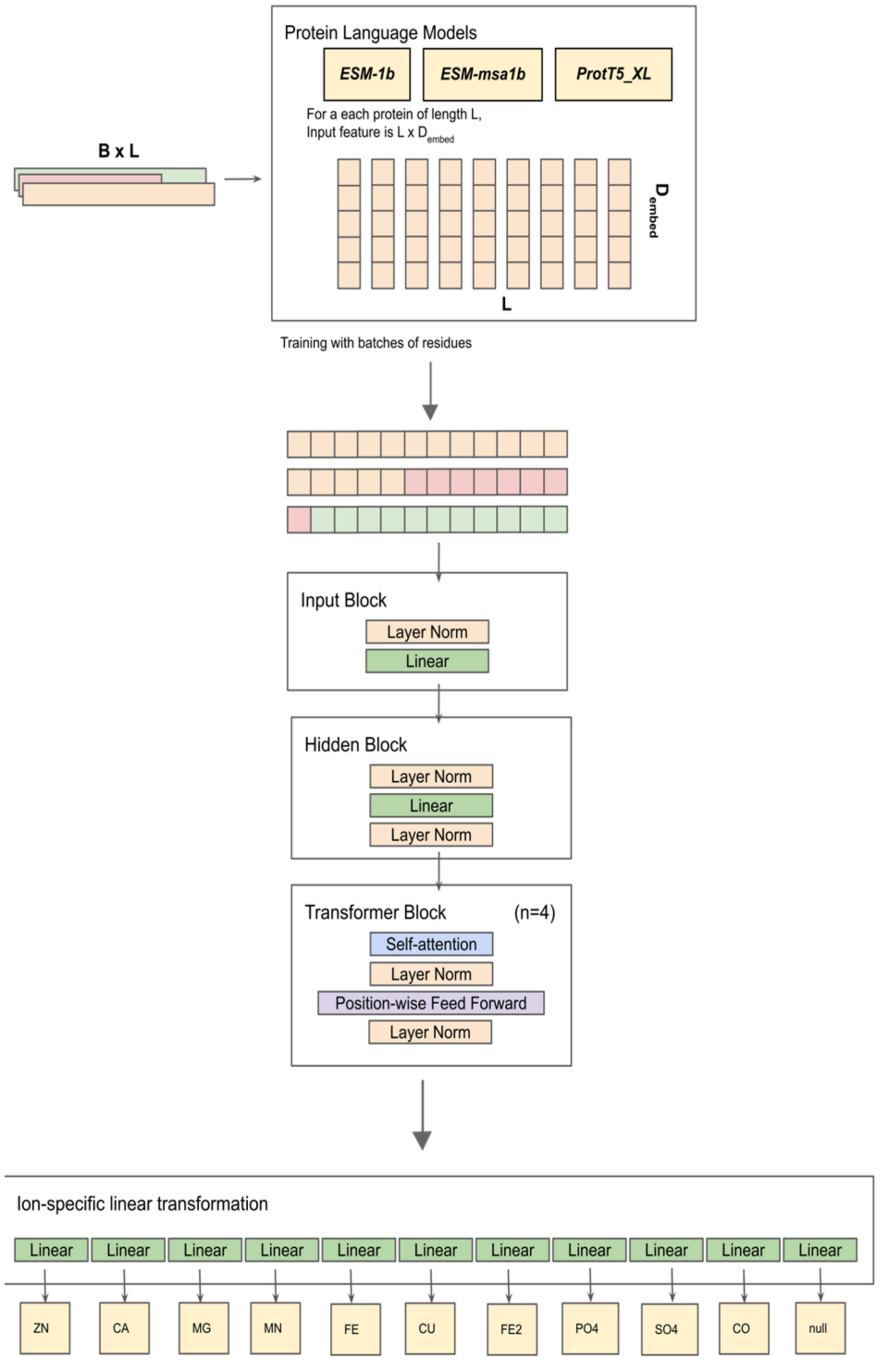
The workflow of M-Ionic consists of an input block, a hidden block, and four transformer encoder blocks. Residues are batched together and trained along the residue-embedding dimension. The final ion-specific linear layers provide output probabilities of multiple ions for each residue.

Each embedding was fed as input to the network one residue at a time. The M-Ionic architecture consists of four blocks: input, hidden, transformer encoder, and ion-specific blocks. It starts with an input block that takes the protein features as input and applies layer normalization, followed by a linear transformation (input dimension = 1280 for ESM-2 or 1024 for ProtT5 and output dimension of the layer = 128) and a Leaky ReLU as the activation function. The hidden block processes the features by applying layer normalization with dropout regularization with probability *P* = .2. This is followed by a linear layer (input and hidden dimension = 128), a Leaky ReLU activation function, and another layer normalization with dimension 128. The next module consists of four encoder layers, each consisting of a self-attention layer and a position-wise feed-forward dense layer. This module (also referred to as the Transformer Block in Fig. 1) is synonymous with the encoder block from the transformer network described in Vaswani *et al.* (2017). Lastly, a linear transformation for each ion (10) and one exclusive dimension (1) for designating the non-binding status of the residue make up the ion-specific block. In this module, a linear transformation is applied to the output of the Transformer Block (input dimension = 128 and output dimension = 1) with the Leaky ReLU activation function. Again, the same linear transformation is applied to the above output, resulting in a vector of dimension Batch Size × Maximum Length for each ion.

In summary, the model utilized self-attention (four attention heads) and feed-forward networks within the transformer architecture to effectively process protein features and make accurate predictions for multiple ions. The model was trained for 30 epochs using the Adam optimiser and BCEWithLogits Loss with a learning rate of 0.001. A learning rate scheduler (PyTorch implementation of StepLR with step size = 10) decayed the learning rate if the training performance did not improve. The output is an *Lx11* dimensional vector with the binding probabilities of multiple metal ions for each residue in a protein of length *L*. The complete model architecture is available on the associated GitHub repository and depicted graphically in Fig. 1.

### 2.4 Evaluation metrics

Precision, recall, F1-score, and Matthew's correlation coefficient (MCC) were used to evaluate how methods perform on metal-binding-site prediction.

$$\begin{array}{l} \mathrm{Precision}\;=\;\frac{\mathrm{TP}}{\mathrm{TP}\;+\;\mathrm{FP}\;}\\ \mathrm{Recall}\;=\;\frac{\mathrm{TP}}{\mathrm{TP}\;+\;\mathrm{FN}}\\ F1\;\mathrm{Score}\;=\;\frac{2\;\times \;\mathrm{Precision}\;\times \;\mathrm{Recall}}{\mathrm{Precision}\;+\;\mathrm{Recall}}\\ M\mathrm{CC}\;=\;\frac{\left(\mathrm{TP}\times \mathrm{TN})\;-\;(\mathrm{FP}\times \mathrm{FN}\right)}{\sqrt{(\mathrm{TP}+\mathrm{FP})\times (\mathrm{TP}+\mathrm{FN})\times (\mathrm{FP}+\mathrm{TN})\times (\mathrm{FN}+\mathrm{TN})\;}} \end{array}$$

TP represents the number of true positives (metal-binding residues predicted correctly), FP represents the number of false positives (non-metal-binding residues predicted as metal binding), TN represents the number of true negatives (non-metal-binding residues predicted correctly), and FN represents the number of false negatives (metal-binding residues predicted as non-metal binding). Additionally, to evaluate protein-level prediction (ability to distinguish between metal-binding and non-binding proteins), we used ROC and reported the area under the ROC curve (AUROC) score. Precision–recall (PR) curves and area under the PR curves (AUPR) were also reported.

## 3 Results and discussion

The evaluation of the metal-binding prediction methods was conducted at two levels: (i) protein level (i.e. whether the method could predict if the protein had a likelihood to bind to metals or not) and (ii) residue level (i.e. the ability to predict the probability of each residue binding to multiple metal ions).

### 3.1 M-Ionic identifies metal-binding proteins

**Figure 2.**
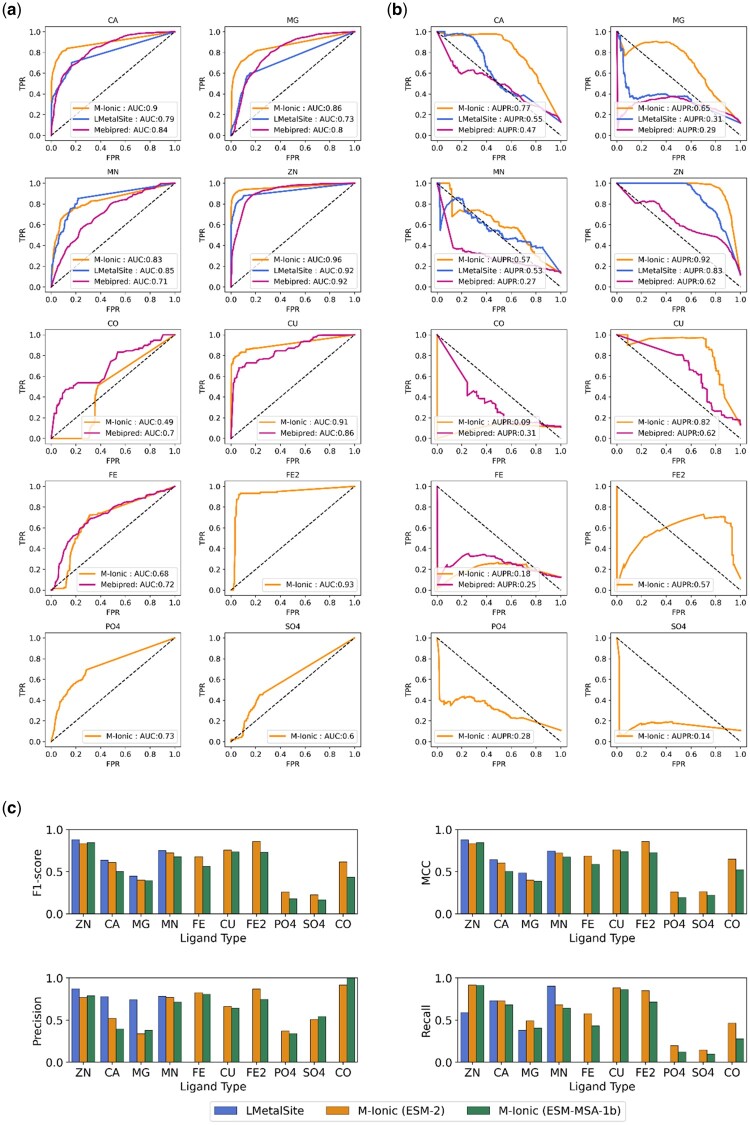
(a) Protein level: Comparison of ROC curves for the performance of each ion type for M-Ionic (this study) trained on ESM-2 embeddings, LMetalSite (Yuan *et al.* 2022b) and mebi-pred (Aptekmann *et al.* 2022) (b) Protein level: Comparison of precision–recall curves for the performance of each ion type for each of the methods (c) Residue level: MCC scores of performance of M-Ionic (trained on ESM-2 and ESM-MSA-1b embeddings) and LMetalSite (Yuan *et al.* 2022b) on ‘Recent BioLip’ dataset (i.e. outside training data).

ROC curves and PR curves were employed to evaluate whether methods could correctly distinguish proteins that bind to metals and those that do not. M-Ionic produces the binding probabilities for each of the 10 metal ions for each residue in the protein. In contrast, mebi-pred (Aptekmann *et al.* 2022) generated only protein-level predictions (i.e. the probability of any protein sequence binding to a metal ion). LMetalSite (Yuan *et al.* 2022b), like M-Ionic, gave binding probabilities at the residue level for four ions (Ca^2+^, Mg^2+^, Mn^2+^, Zn^2+^) as well as 0 or 1 logits indicating the metal-binding status of each residue. The corresponding predicted probabilities for the residues indicated with logit = 1 were used in the ROC curve. LMetalSite and M-Ionic differ in their inputs and how the network is trained. LMetalSite batches proteins of different lengths, pads them together, and trains along the length of the proteins. M-Ionic, however, is trained on the embedding dimension (ie 1280 for ESM-2). Figure 2a and b shows the performance of M-Ionic compared against recent methods mebi-pred (Aptekmann *et al.* 2022) and LMetalSite (Yuan *et al.* 2022b) on the ‘Recent BioLip’ (and the negative dataset). We see that M-Ionic reports a higher true positive rate at false positive rates (except for Co and Fe) compared to other methods and a 30% increase in MCC (Table 1). A similar performance was observed when tested on the independent test set—TestFold6 (and the negative dataset) (Supplementary Table S2 and Supplementary Fig. S1a and b).

### 3.2 M-Ionic identifies metal-binding residues

M-Ionic identifies residues interacting with multiple ions (residue-level prediction) at comparable performance with LMetalSite based on F1-score and MCC (Fig. 2c). M-Ionic has better recall (especially for Zn^2+^ and Mg^2+^) but has lower precision values than LMetalSite. Supplementary Table S3 shows the performance of M-Ionic (trained with ESM-2) with other evaluation metrics. The results from the benchmark set from MIonSite (Qiao and Xie 2019) are reported in Supplementary Table S4, showing that for the eight metal ions compared, M-Ionic performs comparably to the other predictors. A similar performance was observed when the methods were tested on the M-Ionic independent test set—TestFold6 (Supplementary Table S5 and Supplementary Fig. S1c).

### 3.3 Ablation experiments

Three classes of features were used to train the network: (i) Sequence-based (ESM-2, ProtT5), (ii) MSA-based (PSSM, ESM-MSA-1b), and (iii) Structural properties (DSSP). We did not see an improvement in performance using ProtT5 residue-level embeddings (Supplementary Table S5).

#### 3.3.1 Impact of evolutionary information

We observed that certain metal ions, e.g. iron and zinc, have a higher binding affinity to certain amino acids (e.g. cysteine, histidine) (Supplementary Fig. S2), as shown in Barber-Zucker *et al.* (2017) and Cao *et al.* (2017). However, no improvement was observed using explicit evolutionary information to improve the residue-level prediction performance, i.e. ESM-MSA-1b in Fig. 2c. The 22-dimensional PSSM feature vector resulted in a lower performance than the model trained with ESM-2, whereas the 768-dimensional ESM-MSA-1b embedding performed at par with ESM-2. When the MSA-based features were combined with ESM2 features, a slight increase in performance was observed (Supplementary Table S3).

#### 3.3.2 Impact of structural information

Just as with the performance of the one-hot encoding of the protein sequence, the 14-dimensional DSSP vector yielded null performance when trained with the same architecture. We used combinations of the DSSP features with ESM-2 residue embeddings. However, there was a drop in the performance for feature combinations involving structural features (Supplementary Table S5). The M-Ionic architecture (Fig. 1) relies on the information within each residue embedding. The DSSP vector for an individual residue comprises that residue’s secondary structure and bond angles. Since it does not contain context-dependent neighbourhood information, using structural features on a residue level does not yield better results.

### 3.4 Single residue embeddings can predict metal-binding sites

**Figure 3.**
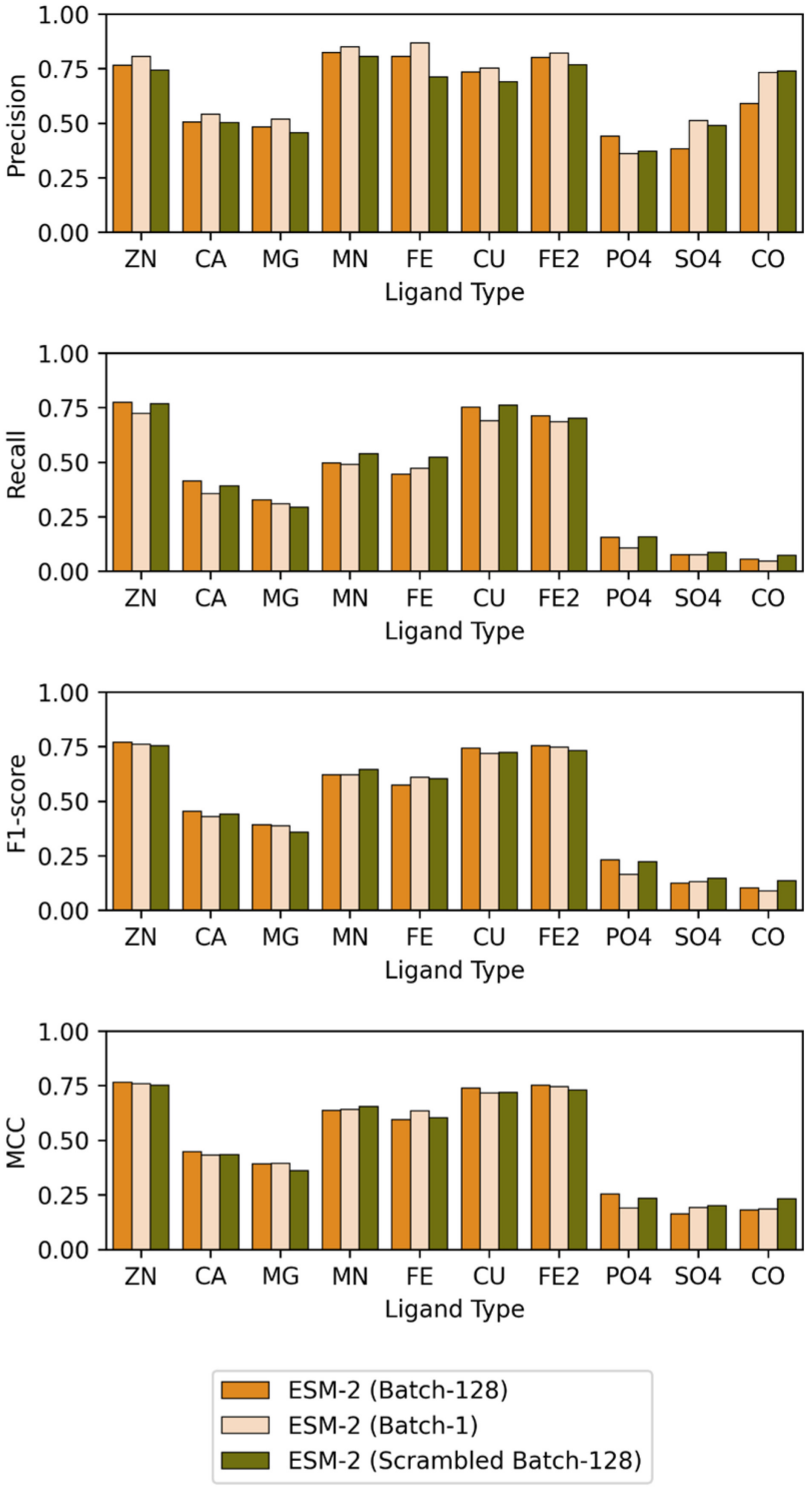
Evaluation metrics (precision, recall, F1, MCC) for the various models trained (a) ESM-2 (Batch-128) is trained with ESM-2 model with batch size 128 as in original M-Ionic implementation (b) ESM-2 (Batch-1) is trained with ESM-2 model with batch size 1 (c) ESM-2 (Scrambled Batch-128) trained on ESM-2 model with batch size 128 but with the residues within each batch were shuffled randomly along the length dimension such that the positions of the residues within the sequence were changed, but the embedding of each residue was untouched.

To verify whether the embeddings from neighbouring residues influenced the prediction of the residue of interest, we retrained the M-Ionic network with batch size 1 (ESM-2 (Batch-1), i.e. the network is trained one-residue at a time) and by scrambling the order of residues within a batch of 128 residues (ESM-2 (Scrambled Batch-128), i.e. scrambling the order of the residues from the original protein sequence). No difference between the models was observed, revealing that the network uses only single residue information (Fig. 3 and Supplementary Table S6). Therefore, M-Ionic shows that a single residue embedding already contains neighbourhood information and is sufficient to predict the metal-binding probability for that residue.

### 3.5 Performance of mutated binding residues

**Figure 4.**
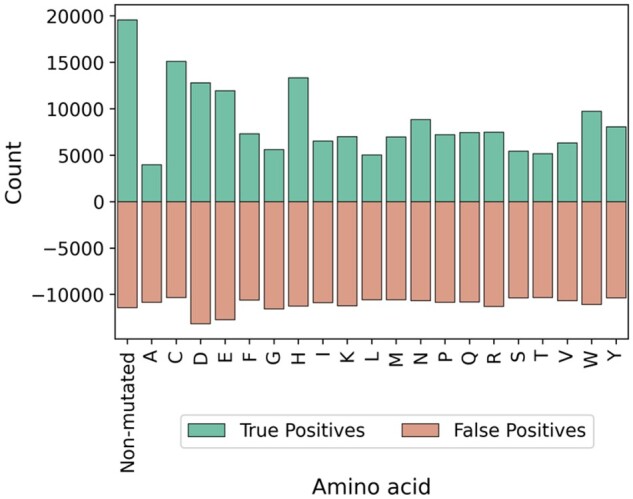
Count plot indicating the number of true and false positives of the M-Ionic predictions. Here, the predictions from the non-mutated sequence are compared against the predictions on the mutated sequence (i.e. when all the binding residues are replaced with one of the amino acids). When an ion-binding is mutated to anything but a His or Cys (mostly from Zn^2+^) and Asp or Glu (mostly from Ca^2+^) most (72.4%) true binding residues are missed. The false positives remain similar for any type of mutation.

To further validate our method, we mutated all the residues involved in metal binding to another amino acid and studied the impact of these mutations on predicted probabilities from M-Ionic. The residues involved in binding were identified from the ‘Recent BioLip’ dataset. Twenty new sequences were generated by replacing all the identified metal-binding residues with one of the 20 amino acids, i.e. one sequence where all the binding residues are mutated with alanine, one sequence where all the binding residues are mutated with leucine, and so on. We found that the number of predicted true positives dropped when metal-binding residues were mutated to neutral residues like alanine or glycine (Fig. 4). In contrast, the number of false positives remained the same on average. Cysteine and histidine mutation, however, show many true positive residues. However, we expect this because those residues are frequently involved in binding (especially for Zn^2+^ and Fe^2+^) (Supplementary Fig. S2). Calculating the number of binding sites per ion associated with each amino acid (Supplementary Table S10), we found that hydrophobic residues such as alanine and leucine are annotated as binding sites in the BioLip database. However, the number of binding sites to these residues is far lower than those of cysteine and histidine. For the protein structures we analysed with an alanine annotated as metal binding, we suspect the ion binds to the protein backbone (e.g. 1BCI). It is to be noted that artefacts in the training data (i.e. annotations from BioLip) could bias this method. Further, the distribution of the output probabilities from M-Ionic for the non-mutated sequence was compared to the output probabilities of the mutated sequence. This analysis can be seen in Supplementary Fig. S3 where all metal-binding residues were replaced with one residue (if the original residue was the same as the residue it was to be mutated to, it was left unchanged), and the output probabilities for only the truly binding ion are considered. The values of various ions were pooled together for Fig. 4 and Supplementary Fig. S3. On comparing the distribution of predicted probabilities of mutated residues, we observe a shift (towards 0.0) from the probabilities of the binding residues of the non-mutated protein sequence. M-Ionic predicts a high binding probability (1.0) for the metal-binding residues in the non-mutated sequence. However, the binding probability of the mutated sequence is lower (Supplementary Fig. S3). Supplementary Fig. S4 shows the number of true positives and false positives for each ion type separately when the metal-binding residues are mutated to one of the 20 amino acids. When metal-binding residues are mutated to non-binding residues, M-Ionic correctly predicts that it is non-binding in 71.7% of binding residues for Zn^2+^ and 63.3% for Ca^2+^ (Zn^2+^ and Ca^2+^ are the most accurately predicted ions). For other ions, M-Ionic is less accurate in its prediction for other ions and correctly identifies less than 50% of mutated binding residues. The fraction (%) of correct prediction of mutated binding residues was obtained by taking the average of true positives when mutated to any one of 20 amino acids and dividing by the number of true positives from non-mutated sequences.

### 3.6 Analysis of M-Ionic performance for specific protein subgroups

The performance of M-Ionic was further analysed on specific groups of proteins (DNA-binding; Transmembrane) and according to different taxonomic groups. No differences between performances within the groups were found (*P*-value >.05 obtained using Mann–Whitney U-test) (Supplementary Tables S–S9).

## 4 Conclusion

On benchmarking against recent proteins and negative set, the performance of M-Ionic was comparable to mebi-pred at the protein level (i.e. the ability to identify which metals a protein binds to). For the most frequent metal ions (Ca^2+^, Mg^2+^, Mn^2+^, Zn^2+^), M-Ionic reported an MCC score of 0.547 compared to 0.49 (LMetalSite) and 0.42 (mebi-pred), respectively. At the residue level (i.e. the ability to predict which residues are involved in metal-ion-specific binding), M-Ionic generated binding-site predictions (with MCC scores comparable with LMetalSite) for six additional metal ions (i.e. Co^2+^, Cu^2+^, ${\text{Po}}_{4}^{3-}$, ${\text{So}}_{4}^{2-}$, Fe^2+^, Fe^3+^). No improvement in performance was observed when ablation experiments with explicit evolutionary and structural features were performed.

This study presents an alternative approach to training pre-trained pLM embeddings by looking within a residue embedding. We show that embeddings from individual residues contain neighbourhood information and have captured long-range dependencies in the pre-training stage. An easy-to-use notebook to generate quick predictions with a probability of binding ten metal ions for each residue in the protein of interest has also been made available.

## Supplementary Material

btad782_Supplementary_Data

## Data Availability

An easy-to-use Google Colab Notebook is freely available at https://bit.ly/40FrRbK. The source code is available from https://github.com/TeamSundar/m-ionic.
