# Supplementary material for: M-Ionic: prediction of metal-ion-binding sites from sequence using residue embeddings
Source: Bioinformatics. 2024 Jan 4;40(1):btad782. doi: 10.1093/bioinformatics/btad782 (PMC10792727; doi:10.1093/bioinformatics/btad782)
Supplement: btad782_Supplementary_Data [file btad782_supplementary_data.pdf]

Supplementary Material

“M-Ionic: Prediction of metal ion binding sites from sequence using residue embeddings.”

Aditi Shenoy[1†], Yogesh Kalakoti[2†], Durai Sundar[2,3], Arne Elofsson[1*]

[1] Science for Life Laboratory and Department of Biochemistry and Biophysics, Stockholm University, 171 21 Solna, Sweden

[2] Department of Biochemical Engineering & Biotechnology, Indian Institute of Technology (IIT) Delhi, New Delhi 110016, India

[3] Yardi School of Artificial Intelligence, Indian Institute of Technology (IIT) Delhi, New Delhi 110016, India

*To whom correspondence should be addressed.

[†]The authors wish it to be known that the first two authors should be regarded as Joint First Authors

Email addresses:
AS: aditi.shenoy@scilifelab.se
YK: yogesh.kalakoti@dbeb.iitd.ac.in
DS: sundar@dbeb.iitd.ac.in
AE: arne@bioinfo.se

Contents:

1. Tables....3

Table S1. Summary of metal-binding proteins in BioLip dataset.... 3

Table S2. Performance on distinguishing metal binding and non-binding proteins using the independent test set generated in this study (TestFold6) and negative set....3

Table S3. Impact of evolutionary (MSA, PSSM) on metal-binding site prediction using 'Recent BioLip' dataset (i.e. independent test set of recent PDB proteins).... 4

Table S4. Benchmark on MIonSite benchmark set....7

Table S5. Impact of structural features (DSSP) on metal-binding site prediction using the independent test set generated in this study (TestFold6).... 9

Table S6. Validating that M-Ionic is trained on the residue level (on the embedding dimension, e.g. 1280 for ESM-2) and not on the protein level (on the length L of the protein) using the independent test set generated in this study (TestFold6)....11

Table S7: Analysis of M-Ionic performance for each ion for each taxon....13

Table S8: Analysis of M-Ionic performance for each ion for DNA-binding proteins.... 14

Table S9: Analysis of M-Ionic performance for each ion for transmembrane against non-membrane proteins....15

Table S10: Number of binding sites for each amino acid associated with each ion in the Recent BioLip proteins dataset....16

2. Figures....17

Figure. S1. Protein- and residue-level comparison on homology reduced independent test set (TestFold6).... 17

(a) Protein-level: Comparison of ROC curves for the performance of each ion type for M-Ionic (this study) trained on ESM-2 embeddings, LMetalSite (Yuan et al., 2022) and mebi-pred (Aptekmann et al., 2022) on homology reduced independent test set (TestFold6) and the negative binding test set (b) Protein-level: Comparison of Precision-Recall curves for the performance of each ion type for each of the methods (c) Residue-level: F1, MCC, Precision and Recall scores of performance of M-Ionic (trained on ESM-2 and ESM-MSA-1b embeddings) and LMetalSite (Yuan et al., 2022) on homology reduced independent test set (TestFold6)....17

Figure. S2. The log odds ratio shows the binding propensity of amino acids for each ion group.
Positive log odds signify that certain amino acids are more likely to bind to that metal group, whereas a negative log odds ratio shows a non-preferential binding....18

Figure. S3. M-Ionic output probabilities distributions (with one plot for each residue type) showing the effect of mutating metal-binding residues.
Firstly, all the metal-binding residues from the original sequences are replaced with another residue one at a time and saved to a new fasta file. So in plot (a), all the binding residues are replaced with alanine (A); in (b), all the binding residues are replaced with Cysteine (C) and so on. These mutated sequences are then used as input to M-Ionic. The output from M-Ionic is the residue-level probabilities of the mutated sequences (represented in blue in the plots) and of the original sequences (represented in orange in the plots). If the original non-mutated sequence is annotated to bind to a particular ion, the output probabilities of the mutated sequences for only that truly binding ion are considered. This means that if the original sequence binds to Zn2+ according to the BioLip annotation, the M-Ionic binding

probability to Zn2+ is considered to plot the distributions in the above plots.........................20
3. References.. 21

1. Tables

Table S1. Summary of metal-binding proteins in BioLip dataset

Ions	Number of protein entries	Number of ions bound with protein receptors	Number of binding residues (P)	Number of non-binding residues (N)	Ratio of binding to non-binding residues (P)/(N)
Zn^{2+}	48593	1952	126278	7704568	0.016
Ca^{2+}	38258	39147	131250	6041662	0.022
Mg^{2+}	34958	5315	78542	6959299	0.011
Mn^{2+}	11650	2850	34315	2254077	0.015
Fe^{3+}	6366	6038	20311	1227819	0.017
Cu^{2+}	5617	27348	16257	948823	0.017
Fe^{2+}	3054	10444	9168	601517	0.015
Co^{2+}	2018	33127	6533	369360	0.018
Po_4^{3-}	2759	2513	12595	605193	0.021
So_4^{2-}	2365	2291	9840	432458	0.023

Table S2. Performance on distinguishing metal binding and non-binding proteins using the independent test set generated in this study (TestFold6) and negative set

Methods	Year	Precision	Recall	Accuracy	F1-score	MCC	AUPR	AUROC
mebi-pred	*2022*	0.206	0.643	0.85	0.311	0.304	0.151	0.752
LMetalSite	*2022*	**0.217**	0.565	**0.87**	**0.314**	0.293	0.146	0.726
M-Ionic	*Current*	0.18	**0.855**	0.787	0.297	**0.329**	**0.161**	**0.819**

**Table S3.** Impact of evolutionary (MSA, PSSM) on metal-binding site prediction using 'Recent BioLip' dataset (i.e. independent test set of recent PDB proteins)							
Ligand Type	Features	Precision	Recall	F1-score	MCC	AUROC	Average Precision
Zn^{2+}	PSSM	0.587	0.818	0.684	0.685	0.902	0.485
	ESM-2 + PSSM	0.759	0.917	0.831	0.830	0.955	0.698
	ESM-2 + ESM-MSA-1b	0.791	0.924	0.852	0.851	0.959	0.733
	ESM-MSA-1b	0.789	0.913	0.846	0.845	0.954	0.722
	ESM-2	0.766	0.915	0.834	0.833	0.954	0.703
	LMetalSite	0.872	0.589	0.880	0.877	0.942	0.777
Ca^{2+}	PSSM	0.317	0.132	0.187	0.193	0.563	0.062
	ESM-2 + PSSM	0.504	0.730	0.596	0.596	0.857	0.374
	ESM-2 + ESM-MSA-1b	0.513	0.722	0.600	0.598	0.853	0.376
	ESM-MSA-1b	0.395	0.681	0.500	0.505	0.828	0.276
	ESM-2	0.521	0.726	0.607	0.605	0.855	0.385
	LMetalSite	0.779	0.730	0.637	0.642	0.768	0.429
Mg^{2+}	PSSM	0.223	0.293	0.253	0.248	0.642	0.072
	ESM-2 + PSSM	0.312	0.494	0.382	0.386	0.742	0.159
	ESM-2 + ESM-MSA-1b	0.290	0.487	0.364	0.369	0.738	0.146
	ESM-MSA-1b	0.377	0.406	0.391	0.385	0.700	0.158
	ESM-2	0.338	0.490	0.400	0.400	0.740	0.170
	LMetalSite	0.740	0.377	0.448	0.485	0.660	0.244
Mn^{2+}	PSSM	0.534	0.262	0.351	0.369	0.630	0.148
	ESM-2 + PSSM	0.742	0.692	0.716	0.714	0.845	0.517

	ESM-2 + ESM-MSA-1b	0.778	0.649	0.708	0.708	0.823	0.509
	ESM-MSA-1b	0.713	0.642	0.676	0.673	0.820	0.462
	ESM-2	0.770	0.680	0.722	0.721	0.839	0.527
	LMetalSite	0.782	0.903	0.749	0.747	0.859	0.566
Fe^{3+}	PSSM	0.783	0.176	0.288	0.368	0.588	0.148
	ESM-2 + PSSM	0.785	0.589	0.673	0.677	0.793	0.468
	ESM-2 + ESM-MSA-1b	0.832	0.538	0.654	0.666	0.769	0.454
	ESM-MSA-1b	0.806	0.431	0.562	0.586	0.715	0.355
	ESM-2	0.825	0.575	0.678	0.686	0.787	0.480
	LMetalSite	N/A	N/A	N/A	N/A	N/A	N/A
Cu^{2+}	PSSM	0.592	0.307	0.404	0.420	0.652	0.192
	ESM-2 + PSSM	0.644	0.903	0.752	0.759	0.948	0.583
	ESM-2 + ESM-MSA-1b	0.688	0.882	0.773	0.775	0.938	0.608
	ESM-MSA-1b	0.641	0.860	0.735	0.739	0.927	0.554
	ESM-2	0.661	0.883	0.756	0.760	0.938	0.586
	LMetalSite	N/A	N/A	N/A	N/A	N/A	N/A
Fe^{2+}	PSSM	0.842	0.345	0.489	0.536	0.672	0.297
	ESM-2 + PSSM	0.796	0.874	0.833	0.832	0.936	0.696
	ESM-2 + ESM-MSA-1b	0.749	0.846	0.795	0.794	0.922	0.636
	ESM-MSA-1b	0.746	0.714	0.730	0.727	0.856	0.535
	ESM-2	0.868	0.849	0.858	0.857	0.924	0.739
	LMetalSite	N/A	N/A	N/A	N/A	N/A	N/A
Co^{2+}	PSSM	0.000	0.000	0.000	0.000	0.500	0.012

	ESM-2 + PSSM	0.845	0.377	0.522	0.561	0.688	0.326
	ESM-2 + ESM-MSA-1b	0.935	0.346	0.505	0.566	0.673	0.331
	ESM-MSA-1b	1.000	0.277	0.434	0.524	0.638	0.286
	ESM-2	0.918	0.464	0.616	0.650	0.732	0.432
	LMetalSite	N/A	N/A	N/A	N/A	N/A	N/A
Po_4^{3-}	PSSM	0.000	0.000	0.000	0.000	0.500	0.017
	ESM-2 + PSSM	0.420	0.209	0.279	0.288	0.602	0.101
	ESM-2 + ESM-MSA-1b	0.404	0.170	0.239	0.254	0.583	0.083
	ESM-MSA-1b	0.341	0.119	0.177	0.194	0.558	0.056
	ESM-2	0.369	0.197	0.256	0.260	0.595	0.086
	LMetalSite	N/A	N/A	N/A	N/A	N/A	N/A
So_4^{2-}	PSSM	0.000	0.000	0.000	0.000	0.500	0.023
	ESM-2 + PSSM	0.513	0.154	0.237	0.272	0.575	0.099
	ESM-2 + ESM-MSA-1b	0.510	0.147	0.229	0.266	0.572	0.095
	ESM-MSA-1b	0.543	0.096	0.163	0.222	0.547	0.073
	ESM-2	0.507	0.145	0.225	0.262	0.571	0.093
	LMetalSite	N/A	N/A	N/A	N/A	N/A	N/A

* Dimension of above embeddings: PSSM (L x 20); ESM-2 (L x 1280); ESM-2 + PSSM (L x 1300); ESM-MSA-1b (L x 768); ESM-2 + ESM-MSA-1b (2048)

**Table S4.** Benchmark on MIonSite benchmark set						
Metal	Method	Year	Sen (%)	Spe (%)	Acc (%)	MCC
Zn^{2+}	MetalDetector	2008	38.26	99.83	98.22	0.565
	S-SITE	2013	45.14	97.88	96.51	0.387
	TargetS	2013	41.70	99.80	98.29	0.588
	COACH	2013	32.39	99.37	97.62	0.422
	IonSeq	2016	70.04	92.56	91.97	0.347
	MIB	2016	40.12	99.07	97.53	0.451
	IonCom	2016	76.72	95.59	95.10	0.474
	MIonSite	2019	70.65	99.68	98.92	0.771
	LMetalSite	2022	78.72	99.81	99.37	0.836
	GASS-Metal (1)	2022	52.14	99.35	98.75	0.515
	GASS-Metal (2)	2022	65.19	99.49	98.96	0.647
	GASS-Metal (3)	2022	75.96	99.68	99.32	0.756
	M-Ionic	Current	76.64	99.50	99.04	0.760
Ca^{2+}	MetalDetector	2008	0.62	99.86	98.03	0.016
	S-SITE	2013	10.17	99.51	97.87	0.159
	TargetS	2013	19.71	99.70	98.23	0.322
	COACH	2013	16.18	97.82	96.32	0.122
	IonSeq	2016	0.00	100	98.16	N/A
	MIB	2016	17.61	99.17	97.68	0.213
	IonCom	2016	28.01	99.47	98.16	0.365
	MIonSite	2019	42.53	99.71	98.66	0.552
	LMetalSite	2022	36.02	99.81	98.97	0.506
	GASS-Metal (1)	2022	9.38	98.84	97.60	0.081
	GASS-Metal (2)	2022	22.93	99.00	97.96	0.219
	GASS-Metal (3)	2022	29.05	99.09	98.15	0.281
	M-Ionic	Current	44.41	99.27	98.45	0.450
Mg^{2+}	MetalDetector	2008	1.57	99.85	98.31	0.043
	S-SITE	2013	35.02	97.28	96.31	0.227
	TargetS	2013	15.16	99.84	98.52	0.298
	COACH	2013	21.12	97.60	96.41	0.144
	IonSeq	2016	0.00	100	98.44	N/A
	MIB	2016	22.21	99.33	98.12	0.268
	IonCom	2016	24.12	99.25	98.07	0.276
	MIonSite	2019	24.35	99.69	98.51	0.362
	LMetalSite	2022	24.88	99.87	99.03	0.410
	GASS-Metal (1)	2022	13.05	99.34	98.75	0.124

	GASS-Metal (2)	2022	33.88	99.54	99.05	0.333
	GASS-Metal (3)	2022	55.74	99.69	99.36	0.550
	M-Ionic	Current	29.01	99.59	98.57	0.380
Mn^{2+}	MetalDetector	2008	21.05	99.72	98.69	0.319
	S-SITE	2013	69.47	98.38	98.01	0.493
	TargetS	2013	28.42	99.75	98.82	0.408
	COACH	2013	27.01	99.82	98.87	0.420
	IonSeq	2016	2.11	99.94	98.67	0.081
	MIB	2016	47.75	99.52	98.84	0.514
	IonCom	2016	54.06	99.39	98.80	0.534
	MIonSite	2019	57.27	99.40	98.84	0.558
	LMetalSite	2022	67.78	99.81	99.41	0.740
	GASS-Metal (1)	2022	19.45	99.45	98.70	0.188
	GASS-Metal (2)	2022	43.75	99.60	99.10	0.433
	GASS-Metal (3)	2022	55.20	99.60	99.35	0.559
	M-Ionic	Current	58.46	99.80	99.29	0.680
Fe^{3+}	MetalDetector	2008	28.57	99.76	99.24	0.360
	S-SITE	2013	90.48	98.78	98.71	0.560
	TargetS	2013	28.57	99.55	99.03	0.296
	COACH	2013	77.52	99.73	99.57	0.725
	IonSeq	2016	80.95	96.85	96.73	0.350
	MIB	2016	52.63	97.83	97.49	0.277
	IonCom	2016	77.12	99.80	99.64	0.756
	MIonSite	2019	82.90	99.75	99.63	0.765
	LMetalSite	2022	95.00	99.93	99.90	0.927
	GASS-Metal (1)	2022	73.40	99.97	99.85	0.733
	GASS-Metal (2)	2022	92.00	99.97	99.85	0.920
	GASS-Metal (3)	2022	92.00	99.97	99.85	0.920
	M-Ionic	Current	80.00	99.78	99.62	0.770
Cu^{2+}	MetalDetector	2008	58.33	99.91	99.47	0.712
	S-SITE	2013	25.00	97.41	96.64	0.138
	COACH	2013	50.00	97.14	96.64	0.268
	IonSeq	2016	41.67	99.11	98.50	0.365
	MIB	2016	83.33	98.03	97.88	0.503
	IonCom	2016	50.00	99.55	99.03	0.517
	MIonSite	2019	48.02	99.63	99.08	0.523
	LMetalSite	2022	81.82	100	99.82	0.904
	GASS-Metal (1)	2022	67.00	99.93	99.85	0.666
	GASS-Metal (2)	2022	67.00	99.93	99.85	0.666

	GASS-Metal (3)	2022	67.00	99.93	99.85	0.666
	M-Ionic	Current	54.55	100	99.56	0.740
Fe^{2+}	MetalDetector	2008	33.33	99.86	99.04	0.496
	S-SITE	2013	66.67	95.54	95.18	0.309
	COACH	2013	66.67	98.05	97.66	0.437
	IonSeq	2016	97.80	99.28	99.26	0.782
	MIB	2016	100	99.44	99.45	0.830
	IonCom	2016	100	99.44	99.45	0.830
	MIonSite	2019	94.98	99.56	99.5	0.830
	LMetalSite	2022	100	100	100	1.000
	GASS-Metal (1)	2022	78.00	99.67	99.67	0.775
	GASS-Metal (2)	2022	89.00	99.67	99.67	0.887
	GASS-Metal (3)	2022	89.00	99.67	99.67	0.887
	M-Ionic	Current	100	100	100	1.000
Co^{2+}	MetalDetector	2008	16.67	99.58	98.69	0.217
	S-SITE	2013	55.21	84.68	84.36	0.113
	COACH	2013	53.21	91.56	91.14	0.162
	MIB	2016	33.33	95.48	94.81	0.138
	MIonSite	2019	58.77	92.58	92.22	0.195
	LMetalSite	2022	22.22	100	99.17	0.469
	GASS-Metal (1)	2022	75.00	99.95	99.90	0.749
	GASS-Metal (2)	2022	75.00	99.95	99.90	0.749
	GASS-Metal (3)	2022	75.00	99.95	99.90	0.749
	M-Ionic	Current	0	100	98.60	0

*GASS-Metal (1) = top 10 results; GASS-Metal (2) = top 100 results; GASS-Metal (3) = all results from search

Table S5. Impact of structural features (DSSP) on metal-binding site prediction using the independent test set generated in this study (TestFold6)

Ligand Type	Features	Precision	Recall	F1-score	MCC	AUROC	Average Precision
Zn^{2+}	DSSP	0.190	0.037	0.062	0.078	0.517	0.021
	ESM2+DSSP	0.678	0.798	0.733	0.732	0.896	0.544
	LMetalSite	0.856	0.780	0.816	0.814	0.889	0.671
	ESM-2	0.739	0.869	0.799	0.798	0.932	0.645
	ESM-MSA-1b	0.712	0.825	0.764	0.763	0.910	0.590
	ProtT5-XL	0.600	0.200	0.300	0.333	0.597	0.150

Ca^{2+}	DSSP	0.106	0.037	0.055	0.053	0.516	0.021
	ESM2+DSSP	0.392	0.430	0.410	0.400	0.709	0.179
	LMetalSite	0.815	0.364	0.504	0.540	0.681	0.308
	ESM-2	0.527	0.549	0.538	0.529	0.770	0.297
	ESM-MSA-1b	0.267	0.435	0.331	0.326	0.707	0.126
	ProtT5-XL	0.000	0.000	0.000	-0.003	0.499	0.005
Mg^{2+}	DSSP	0.121	0.008	0.016	0.029	0.504	0.013
	ESM2+DSSP	0.432	0.337	0.379	0.375	0.666	0.154
	LMetalSite	0.733	0.281	0.406	0.450	0.640	0.214
	ESM-2	0.482	0.434	0.457	0.451	0.714	0.216
	ESM-MSA-1b	0.434	0.327	0.373	0.370	0.661	0.150
	ProtT5-XL	1.000	0.400	0.571	0.629	0.700	0.410
Mn^{2+}	DSSP	0.000	0.000	0.000	0.000	0.500	0.013
	ESM2+DSSP	0.763	0.582	0.660	0.663	0.790	0.450
	LMetalSite	0.830	0.715	0.768	0.768	0.856	0.598
	ESM-2	0.781	0.726	0.753	0.750	0.862	0.571
	ESM-MSA-1b	0.729	0.595	0.655	0.654	0.796	0.439
	ProtT5-XL	0.500	0.500	0.500	0.485	0.742	0.265
Fe^{3+}	DSSP	0.000	0.000	0.000	0.000	0.500	0.022
	ESM2+DSSP	0.776	0.612	0.684	0.683	0.804	0.483
	LMetalSite	N/A	N/A	N/A	N/A	N/A	N/A
	ESM-2	0.755	0.862	0.805	0.802	0.928	0.653
	ESM-MSA-1b	0.748	0.384	0.507	0.529	0.690	0.300
	ProtT5-XL	0.667	0.333	0.444	0.465	0.665	0.233
Cu^{2+}	DSSP	0.000	0.000	0.000	0.000	0.500	0.016
	ESM2+DSSP	0.509	0.711	0.593	0.594	0.850	0.366
	LMetalSite	N/A	N/A	N/A	N/A	N/A	N/A
	ESM-2	0.709	0.835	0.767	0.766	0.915	0.595
	ESM-MSA-1b	0.581	0.790	0.670	0.672	0.890	0.462
	ProtT5-XL	0.600	0.750	0.667	0.667	0.872	0.453
Fe^{2+}	DSSP	0.000	0.000	0.000	0.000	0.500	0.015
	ESM2+DSSP	0.727	0.755	0.741	0.737	0.875	0.553

	LMetalSite	N/A	N/A	N/A	N/A	N/A	N/A
	ESM-2	0.798	0.691	0.741	0.739	0.844	0.556
	ESM-MSA-1b	0.765	0.798	0.781	0.778	0.897	0.614
	ProtT5-XL	0.889	1.000	0.941	0.937	0.993	0.889
Co^{2+}	DSSP	0.000	0.000	0.000	0.000	0.500	0.016
	ESM2+DSSP	0.615	0.037	0.070	0.149	0.518	0.039
	LMetalSite	N/A	N/A	N/A	N/A	N/A	N/A
	ESM-2	0.800	0.264	0.397	0.456	0.632	0.223
	ESM-MSA-1b	0.706	0.053	0.098	0.190	0.526	0.052
	ProtT5-XL	0.400	1.000	0.571	0.630	0.996	0.400
Po_4^{3-}	DSSP	0.000	0.000	0.000	0.000	0.500	0.019
	ESM2+DSSP	0.410	0.244	0.306	0.306	0.618	0.114
	LMetalSite	N/A	N/A	N/A	N/A	N/A	N/A
	ESM-2	0.433	0.347	0.385	0.377	0.669	0.162
	ESM-MSA-1b	0.396	0.186	0.253	0.262	0.590	0.089
	ProtT5-XL	0.000	0.000	0.000	0.000	0.500	0.005
So_4^{2-}	DSSP	0.000	0.000	0.000	0.000	0.500	0.018
	ESM2+DSSP	0.392	0.124	0.189	0.213	0.560	0.064
	LMetalSite	N/A	N/A	N/A	N/A	N/A	N/A
	ESM-2	0.528	0.254	0.343	0.358	0.625	0.148
	ESM-MSA-1b	0.520	0.098	0.166	0.221	0.548	0.068
	ProtT5-XL	0.000	0.000	0.000	0.000	0.500	0.020

Table S6. Validating that M-Ionic is trained on the residue level (on the embedding dimension, e.g. 1280 for ESM-2) and not on the protein level (on the length L of the protein) using the independent test set generated in this study (TestFold6)

Ligand Type	Features	Precision	Recall	F1-score	MCC	AUROC	Average Precision
Zn^{2+}	ESM-2 (Batch-1)	0.806	0.724	0.763	0.760	0.861	0.588
	ESM-2 (Batch-128) *(Base model)*	0.765	0.776	0.771	0.767	0.886	0.598

	ESM-2 (Scrambled Batch-128)	0.744	0.769	0.756	0.753	0.883	0.576
Ca^{2+}	ESM-2 (Batch-1)	0.542	0.357	0.431	0.432	0.676	0.205
	ESM-2 (Batch-128) *(Base model)*	0.505	0.415	0.456	0.449	0.704	0.220
	ESM-2 (Scrambled Batch-128)	0.504	0.392	0.441	0.436	0.693	0.208
Mg^{2+}	ESM-2 (Batch-1)	0.519	0.310	0.388	0.395	0.653	0.169
	ESM-2 (Batch-128) *(Base model)*	0.484	0.329	0.392	0.393	0.662	0.167
	ESM-2 (Scrambled Batch-128)	0.457	0.296	0.359	0.361	0.646	0.144
Mn^{2+}	ESM-2 (Batch-1)	0.852	0.490	0.622	0.642	0.744	0.424
	ESM-2 (Batch-128) *(Base model)*	0.825	0.498	0.621	0.637	0.748	0.417
	ESM-2 (Scrambled Batch-128)	0.807	0.539	0.646	0.656	0.768	0.441
Fe^{3+}	ESM-2 (Batch-1)	0.868	0.472	0.611	0.635	0.735	0.421
	ESM-2 (Batch-128) *(Base model)*	0.807	0.447	0.575	0.594	0.722	0.373
	ESM-2 (Scrambled Batch-128)	0.713	0.524	0.604	0.604	0.760	0.384
Cu^{2+}	ESM-2 (Batch-1)	0.754	0.690	0.720	0.717	0.843	0.525
	ESM-2 (Batch-128) *(Base model)*	0.735	0.753	0.744	0.740	0.874	0.558
	ESM-2 (Scrambled Batch-128)	0.691	0.761	0.724	0.721	0.878	0.530

Fe^{2+}	ESM-2 (Batch-1)	0.822	0.686	0.748	0.748	0.842	0.568
	ESM-2 (Batch-128) *(Base model)*	0.802	0.713	0.755	0.753	0.855	0.576
	ESM-2 (Scrambled Batch-128)	0.767	0.702	0.733	0.730	0.849	0.543
Co^{2+}	ESM-2 (Batch-1)	0.733	0.048	0.091	0.186	0.524	0.051
	ESM-2 (Batch-128) *(Base model)*	0.591	0.057	0.104	0.181	0.528	0.049
	ESM-2 (Scrambled Batch-128)	0.739	0.075	0.136	0.232	0.537	0.070
Po_4^{3-}	ESM-2 (Batch-1)	0.362	0.108	0.166	0.190	0.552	0.056
	ESM-2 (Batch-128) *(Base model)*	0.442	0.157	0.232	0.255	0.577	0.085
	ESM-2 (Scrambled Batch-128)	0.373	0.159	0.223	0.234	0.577	0.075
So_4^{2-}	ESM-2 (Batch-1)	0.513	0.076	0.132	0.192	0.537	0.056
	ESM-2 (Batch-128) *(Base model)*	0.385	0.076	0.127	0.164	0.537	0.046
	ESM-2 (Scrambled Batch-128)	0.489	0.087	0.148	0.201	0.543	0.060

Table S7: Analysis of M-Ionic performance for each ion for each taxon

Ligand Type	Taxonomy	Precision	Recall	F1-score	MCC	AUROC
Zn^{2+}	Archaea	0.734	0.972	0.814	0.827	0.978
	Bacteria	0.664	0.832	0.715	0.725	0.909
	Eukaryota	0.829	0.942	0.870	0.871	0.964
	Viruses	0.623	0.815	0.687	0.695	0.901
Ca^{2+}	Archaea	0.383	0.708	0.428	0.447	0.817
	Bacteria	0.335	0.433	0.358	0.361	0.709

	Eukaryota	0.520	0.696	0.560	0.565	0.829
	Viruses	0.372	0.588	0.436	0.445	0.782
Mg^{2+}	Archaea	0.321	0.591	0.380	0.404	0.784
	Bacteria	0.301	0.513	0.356	0.371	0.749
	Eukaryota	0.421	0.665	0.487	0.509	0.828
	Viruses	0.416	0.594	0.443	0.469	0.795
Mn^{2+}	Archaea	0.783	1.000	0.878	0.883	0.998
	Bacteria	0.680	0.633	0.625	0.638	0.816
	Eukaryota	0.634	0.597	0.581	0.594	0.797
	Viruses	0.665	0.867	0.729	0.740	0.927
Fe^{3+}	Archaea	0.690	0.521	0.551	0.571	0.760
	Bacteria	0.591	0.576	0.550	0.564	0.787
	Eukaryota	0.691	0.665	0.651	0.659	0.831
Cu^{2+}	Bacteria	0.648	0.859	0.721	0.731	0.925
	Eukaryota	0.625	0.799	0.679	0.692	0.896
Fe^{2+}	Bacteria	0.813	0.826	0.768	0.790	0.911
	Eukaryota	0.920	0.783	0.815	0.831	0.891
Co^{2+}	Bacteria	0.445	0.292	0.338	0.351	0.646
	Eukaryota	0.389	0.468	0.421	0.424	0.733
Po_4^{3-}	Archaea	0.399	0.167	0.229	0.246	0.581
	Bacteria	0.224	0.190	0.187	0.187	0.591
	Eukaryota	0.246	0.196	0.203	0.205	0.595
	Viruses	0.150	0.117	0.124	0.120	0.555
So_4^{2-}	Archaea	0.204	0.122	0.131	0.134	0.552
	Bacteria	0.165	0.109	0.119	0.122	0.553
	Eukaryota	0.157	0.115	0.113	0.118	0.556
	Viruses	0.063	0.025	0.036	0.039	0.512

Table S8: Analysis of M-Ionic performance for each ion for DNA-binding proteins

Ligand Type	Type	Precision	Recall	F1-score	MCC	AUROC	Average Precision
Zn^{2+} (p-value = 0.0048)	non-binding	0.780	0.911	0.824	0.828	0.949	0.758
	DNA-binding	0.867	0.980	0.913	0.913	0.985	0.863
Ca^{2+} (p-value = 0.7264)	non-binding	0.451	0.606	0.487	0.492	0.788	0.390
	DNA-binding	0.500	0.333	0.400	0.403	0.665	0.342
Mg^{2+} (p-value = 0.6854)	non-binding	0.371	0.599	0.430	0.450	0.794	0.332
	DNA-binding	0.307	0.551	0.370	0.395	0.773	0.304

Mn^{2+} (p-value = 1)	non-binding	0.660	0.647	0.620	0.633	0.822	0.540
	DNA-binding	0.800	0.800	0.800	0.793	0.897	0.647
Fe^{3+} (p-value = N/A)	non-binding	0.629	0.599	0.581	0.593	0.798	0.519
	DNA-binding	-	-	-	-	-	-
Cu^{2+} (p-value = N/A)	non-binding	0.638	0.834	0.704	0.715	0.913	0.605
	DNA-binding	-	-	-	-	-	-
Fe^{2+} (p-value = 0.3420)	non-binding	0.862	0.801	0.786	0.806	0.899	0.712
	DNA-binding	1.000	1.000	1.000	1.000	1.000	1.000
Co^{2+} (p-value = 0.3914)	non-binding	0.424	0.395	0.391	0.399	0.697	0.346
	DNA-binding	0.000	0.000	0.000	0.000	0.500	0.010
Po_4^{3-} (p-value = 0.6437)	non-binding	0.242	0.191	0.197	0.198	0.592	0.147
	DNA-binding	0.077	0.333	0.125	0.148	0.657	0.100
So_4^{2-} (p-value = 0.0878)	non-binding	0.159	0.108	0.113	0.117	0.552	0.104
	DNA-binding	0.000	0.000	0.000	-0.019	0.497	0.070

Table S9: Analysis of M-Ionic performance for each ion for transmembrane against non-membrane proteins

Ligand Type	Type	Precision	Recall	F1-score	MCC	AUROC	Average Precision
Zn^{2+} (p-value = 0.0054)	Transmembrane	0.791	0.919	0.836	0.839	0.953	0.771
	Non-membrane	0.692	0.851	0.741	0.750	0.920	0.664
Ca^{2+} (p-value = 0.2037)	Transmembrane	0.436	0.605	0.478	0.483	0.786	0.381
	Non-membrane	0.510	0.605	0.521	0.525	0.791	0.425
Mg^{2+} (p-value = 0.6428)	Transmembrane	0.369	0.595	0.428	0.447	0.791	0.331
	Non-membrane	0.366	0.629	0.436	0.462	0.811	0.330
Mn^{2+} (p-value = 0.2683)	Transmembrane	0.666	0.660	0.630	0.643	0.828	0.551
	Non-membrane	0.597	0.500	0.513	0.528	0.749	0.411
Fe^{3+} (p-value = 0.5236)	Transmembrane	0.624	0.589	0.572	0.584	0.793	0.510
	Non-membrane	0.656	0.656	0.632	0.643	0.828	0.567
Cu^{2+} (p-value = 0.0369)	Transmembrane	0.617	0.845	0.695	0.707	0.918	0.585
	Non-membrane	0.726	0.789	0.741	0.747	0.893	0.691
Fe^{2+} (p-value = 0.3459)	Transmembrane	0.884	0.823	0.807	0.827	0.910	0.735
	Non-membrane	0.667	0.625	0.617	0.630	0.812	0.544
Co^{2+} (p-value = 0.11805)	Transmembrane	0.442	0.413	0.408	0.416	0.706	0.361
	Non-membrane	0.000	0.000	0.000	0.000	0.500	0.010
Po_4^{3-} (p-value = 0.3561)	Transmembrane	0.243	0.197	0.201	0.202	0.595	0.151
	Non-membrane	0.190	0.085	0.101	0.109	0.541	0.066

So_4^{2-} (p-value = 0.3023)	Transmembrane	0.158	0.111	0.115	0.118	0.553	0.106
	Non-membrane	0.150	0.053	0.079	0.089	0.527	0.077

Table S10: Number of binding sites for each amino acid associated with each ion in the Recent BioLip proteins dataset

Ion / Amino acid	Ca^{2+}	Co^{2+}	Cu^{2+}	Fe^{3+}	Fe^{2+}	Mg^{2+}	Mn^{2+}	Po_4^{3-}	So_4^{2-}	Zn^{2+}
A	318	2	9	8	3	1348	29	145	72	22
C	46	57	137	106	14	80	20	40	17	17568
D	6121	80	48	381	112	3100	1135	235	54	3392
E	2478	142	37	734	102	2370	545	227	79	1309
F	225	6	6	1	4	750	1	66	46	19
G	1429	6	27	5	1	2958	60	716	217	25
H	236	262	1306	1023	751	1351	801	329	117	8723
I	365	4	1	8	27	748	46	80	19	8
K	229	7	6	17	1	2659	27	462	185	95
L	327	0	0	1	0	1230	2	86	68	19
M	120	18	67	0	6	334	68	37	29	10
N	1566	8	8	38	19	1452	91	298	111	67
P	242	0	0	0	1	992	39	73	77	31
Q	549	19	2	25	10	665	23	107	68	44
R	247	6	12	36	2	3953	23	857	416	40
S	718	14	2	3	0	1390	74	411	160	44
T	877	6	0	1	4	1985	9	256	123	43
V	411	0	3	0	0	1497	5	71	54	13
W	136	0	0	8	2	310	0	44	51	10
Y	396	0	16	36	3	939	18	190	95	42

2. Figures

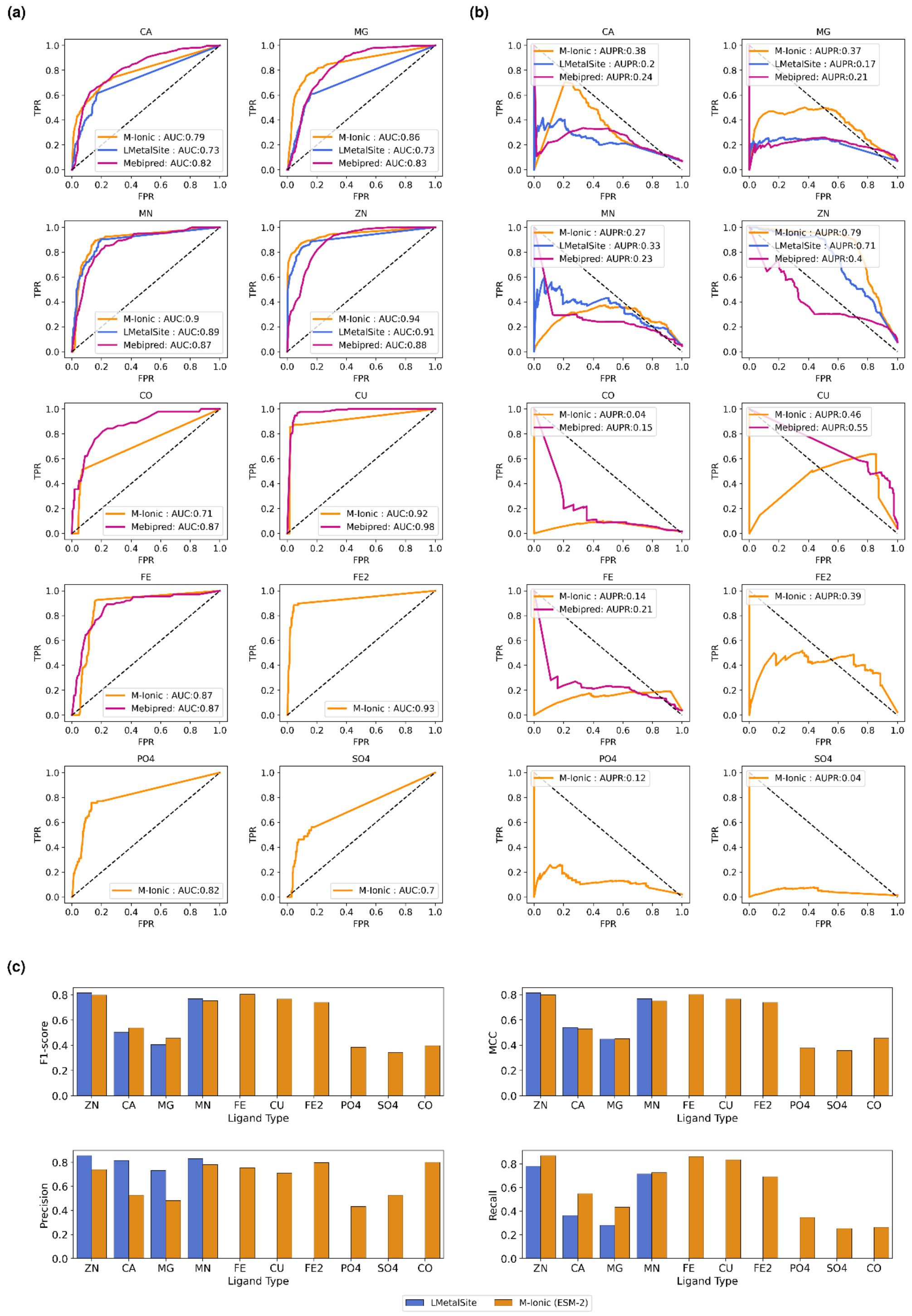

Figure. S1. Protein- and residue-level comparison on homology reduced independent test set (TestFold6)

(a) Protein-level: Comparison of ROC curves for the performance of each ion type for M-Ionic (this study) trained on ESM-2 embeddings, LMetalSite (Yuan et al., 2022) and mebi-pred (Aptekmann et al., 2022) on homology reduced independent test set (TestFold6) and the negative binding test set (b) Protein-level: Comparison of Precision-Recall curves for the performance of each ion type for each of the methods (c) Residue-level: F1, MCC, Precision and Recall scores of performance of M-Ionic (trained on ESM-2 and ESM-MSA-1b embeddings) and LMetalSite (Yuan et al., 2022) on homology reduced independent test set (TestFold6).

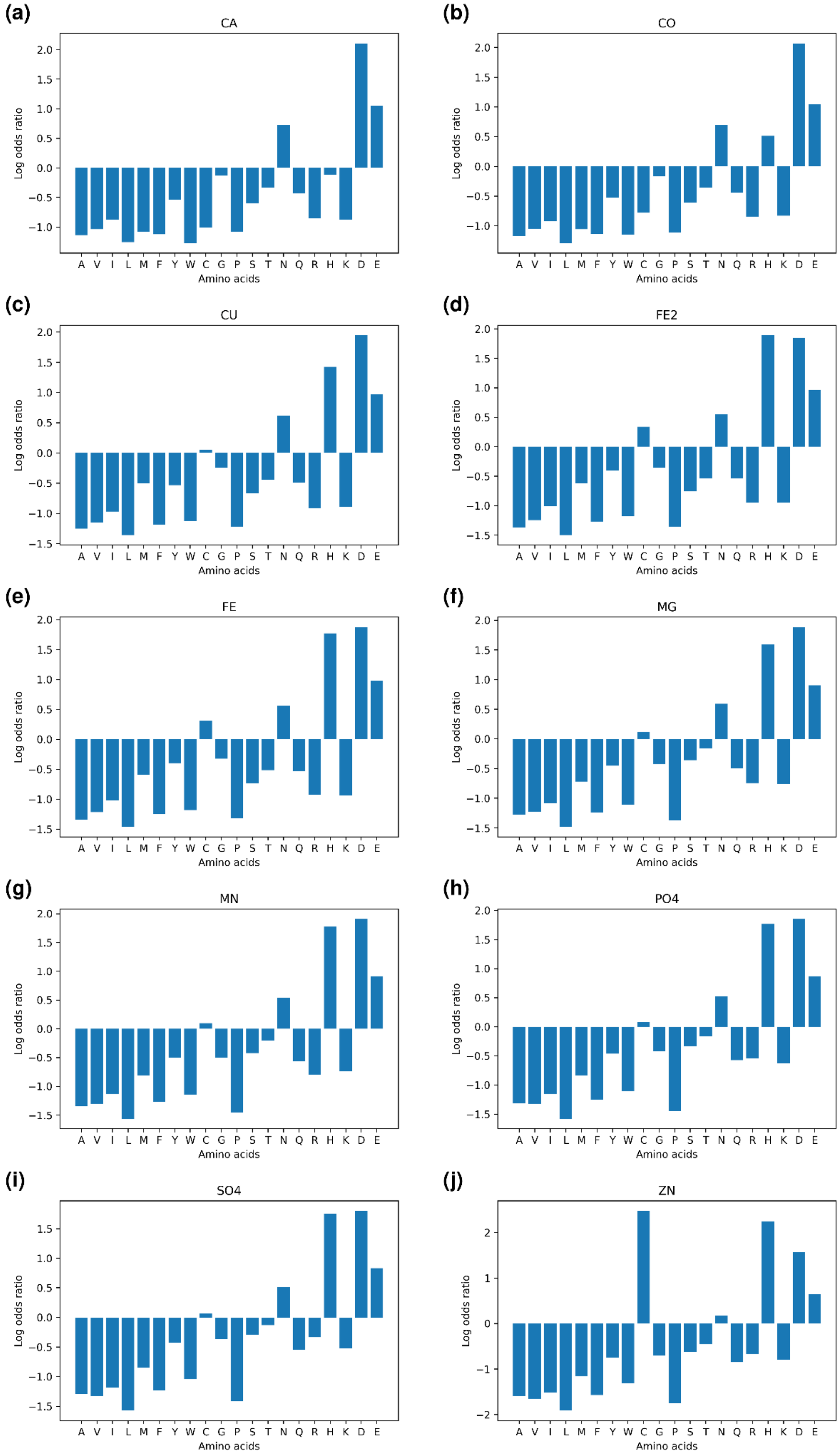

Figure. S2. The log odds ratio shows the binding propensity of amino acids for each ion group.
Positive log odds signify that certain amino acids are more likely to bind to that metal group, whereas a negative log odds ratio shows a non-preferential binding.

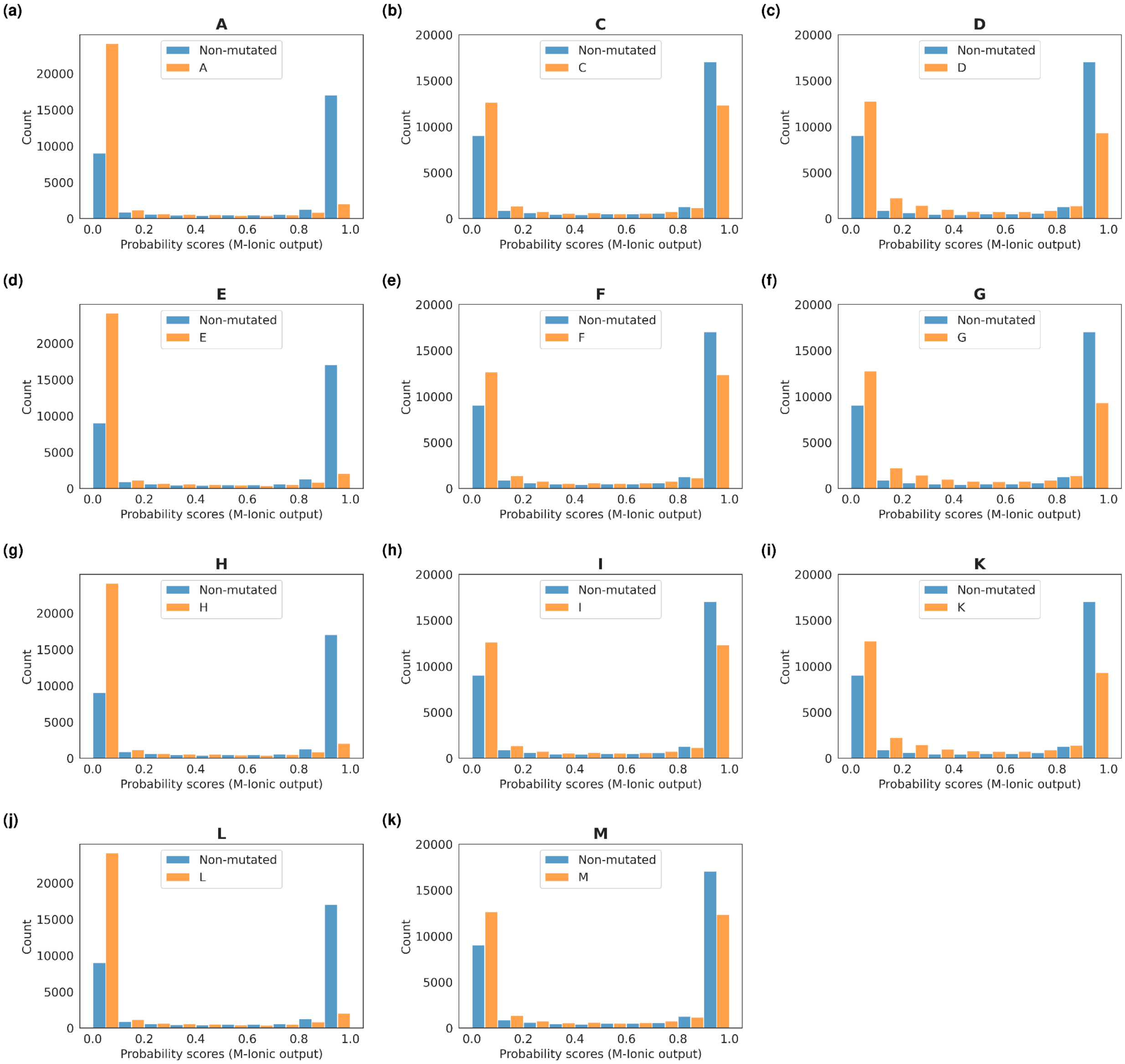
(a)
A
Non-mutated
A
Count
Probability scores (M-Ionic output)
(b)
C
Non-mutated
C
Count
Probability scores (M-Ionic output)
(c)
D
Non-mutated
D
Count
Probability scores (M-Ionic output)
(d)
E
Non-mutated
E
Count
Probability scores (M-Ionic output)
(e)
F
Non-mutated
F
Count
Probability scores (M-Ionic output)
(f)
G
Non-mutated
G
Count
Probability scores (M-Ionic output)
(g)
H
Non-mutated
H
Count
Probability scores (M-Ionic output)
(h)
I
Non-mutated
I
Count
Probability scores (M-Ionic output)
(i)
K
Non-mutated
K
Count
Probability scores (M-Ionic output)
(j)
L
Non-mutated
L
Count
Probability scores (M-Ionic output)
(k)
M
Non-mutated
M
Count
Probability scores (M-Ionic output)
0
5000
10000
15000
20000
0.0
0.2
0.4
0.6
0.8
1.0

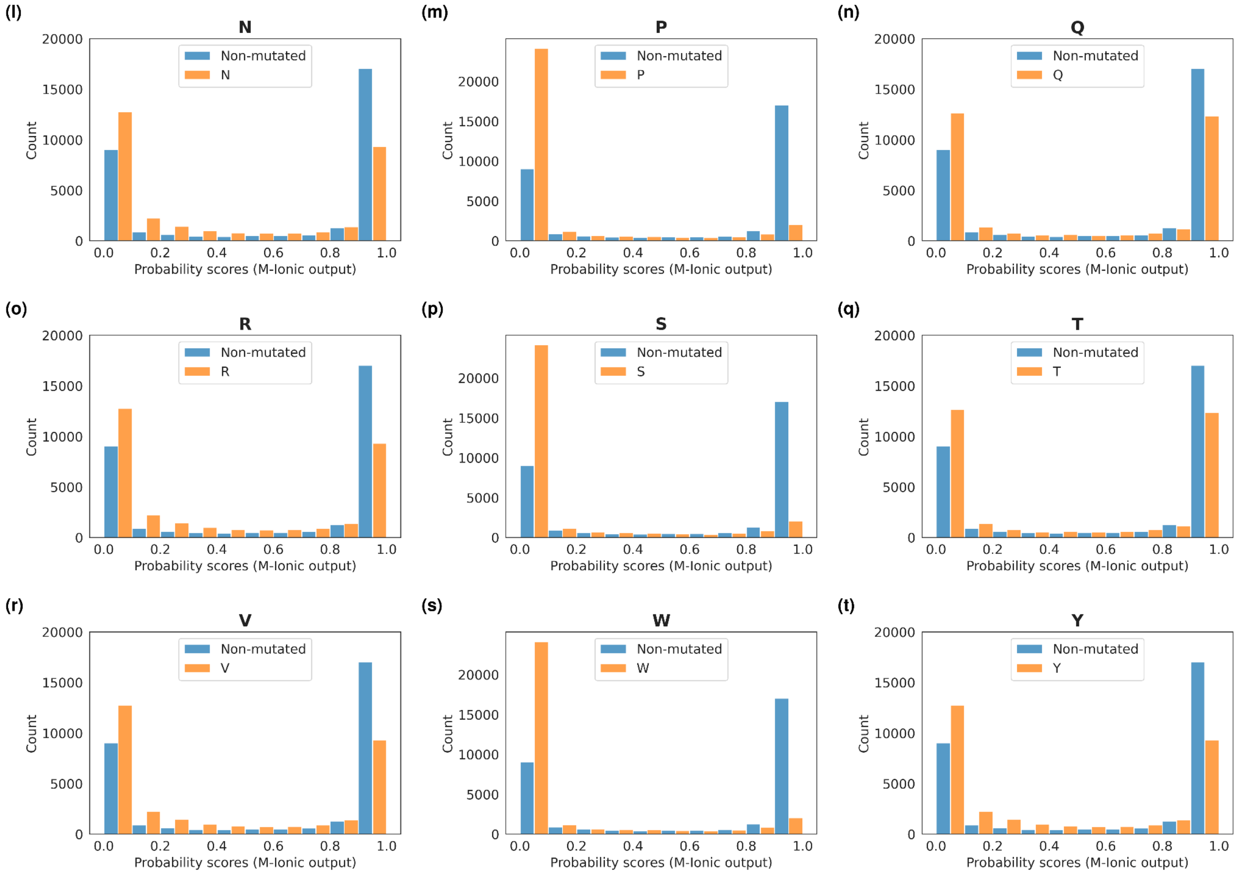

Figure. S3. M-Ionic output probabilities distributions (with one plot for each residue type) showing the effect of mutating metal-binding residues.

To examine the ability of the method to identify the effect of mutations, we systematically replaced all metal-binding residues with one of the 20 amino acids one at a time. In plot (a), all the binding residues are replaced with alanine (A); in (b), all the binding residues are replaced with Cysteine (C) and so on. These mutated sequences are then used as input to M-Ionic, and the binding probabilities for each of the residues are obtained. If the original residue is annotated to bind to a particular ion, the output probabilities of the mutated sequences for only that truly binding ion are considered. This means that if the original sequence binds to Zn^{2+}, the M-Ionic binding probability to Zn^{2+} is considered, and the probabilities for other ions are ignored. In the plots, the output probabilities from M-Ionic for the mutated residue (in orange) and original residue (in blue) are shown. We have pooled the data for all ions into single plots per residue type.

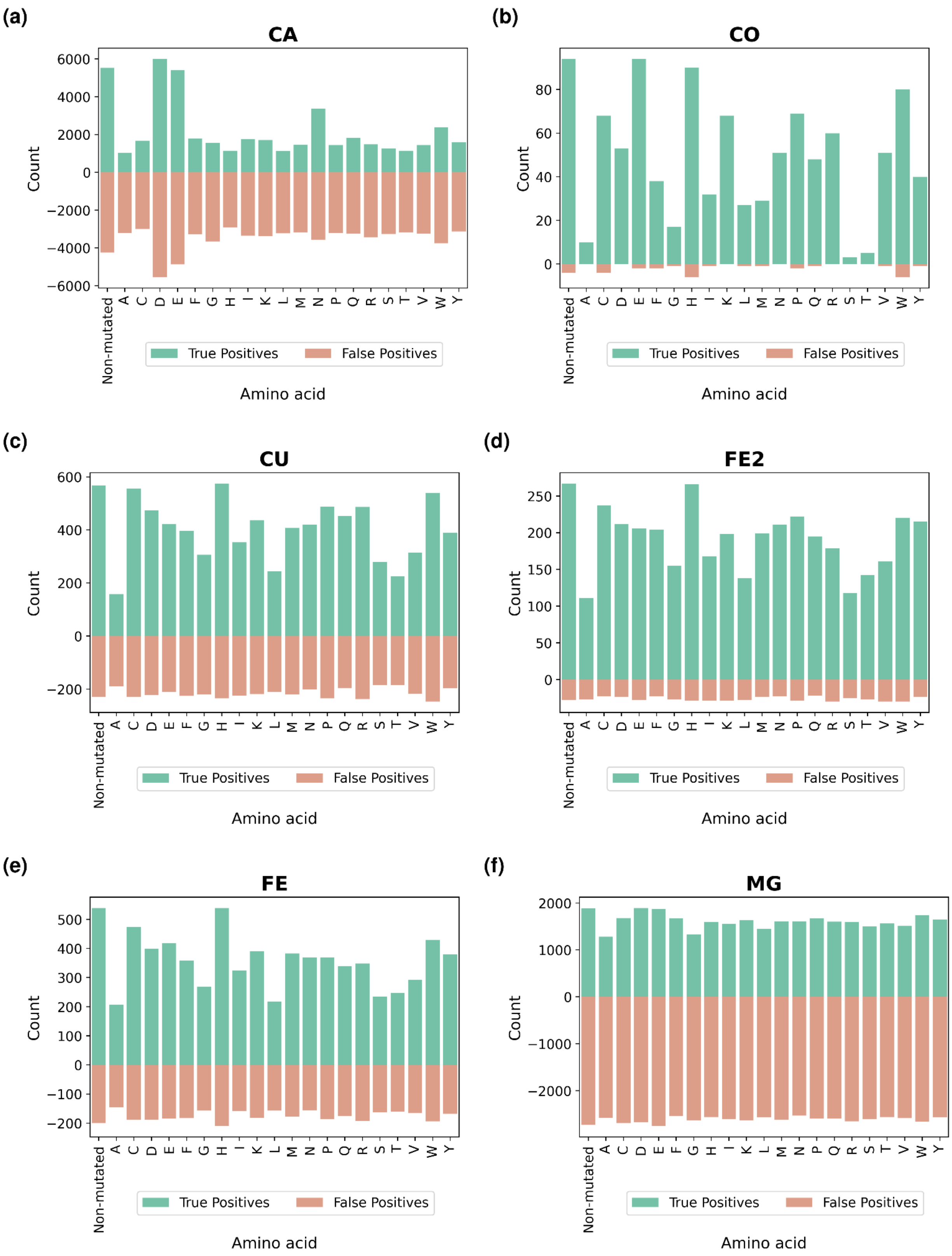
(a)
CA
(b)
CO
(c)
CU
(d)
FE2
(e)
FE
(f)
MG
Count
Amino acid
Non-mutated
A C D E F G H I K L M N P Q R S T V W Y
True Positives
False Positives

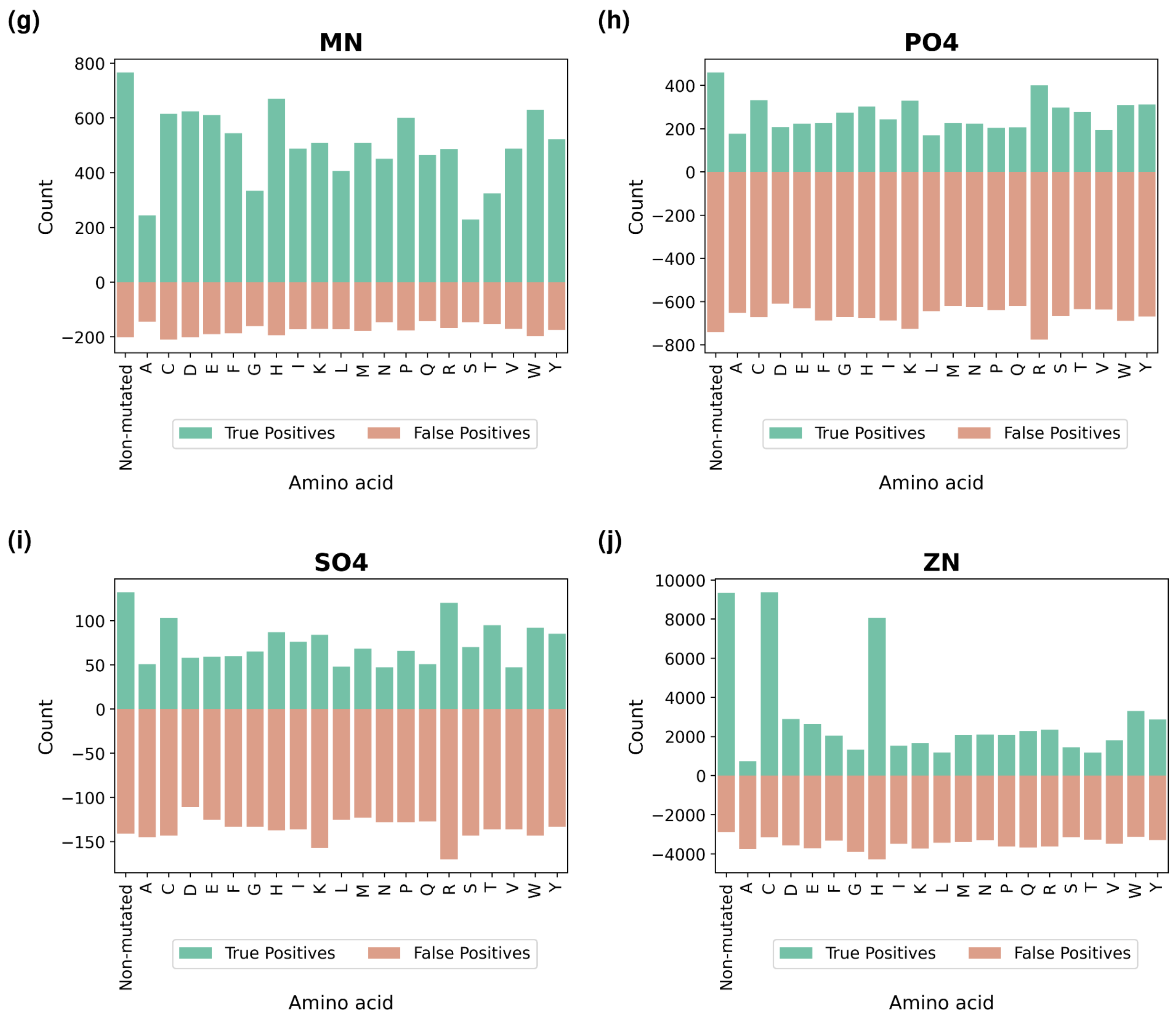

Figure. S4. Count plot for each ion type indicating the number of true and false positives of the M-Ionic predictions of mutated metal-binding residues.

To examine the effect of mutating residues on prediction performance with respect to each ion type individually, each of the above plots shows the count of the number of true positives (TPs) and false positives (FPs) when the metal-binding residues are mutated to one of the 20 amino acid types. Plot (a) shows the TPs and FPs for Calcium; (b) shows the TPs and FPs for Cobalt and so on. The amino acids on the x-axis represent the residue all the metal binding residues were mutated to. In this figure, the values are not pooled as in Figure 4 and Figure S3.

3. References

Aptekmann, A. A., Buongiorno, J., Giovannelli, D., Glamoclija, M., Ferreiro, D. U., & Bromberg, Y. (2022). mebipred: Identifying metal-binding potential in protein sequence. *Bioinformatics*, *38*(14), 3532–3540. https://doi.org/10.1093/bioinformatics/btac358

Yuan, Q., Chen, S., Wang, Y., Zhao, H., & Yang, Y. (2022). Alignment-free metal ion-binding site prediction from protein sequence through pretrained language model and multi-task learning. *Briefings in Bioinformatics*, *23*(6), bbac444. https://doi.org/10.1093/bib/bbac444
